# Effects of *N*‐acetyl‐seryl‐asparyl‐lysyl‐proline on blood pressure, renal damage, and mortality in systemic lupus erythematosus

**DOI:** 10.14814/phy2.13084

**Published:** 2017-01-26

**Authors:** Pablo Nakagawa, Juan X. Masjoan‐Juncos, Heba Basha, Branislava Janic, Morel E. Worou, Tang‐Dong Liao, Cesar A. Romero, Edward L. Peterson, Oscar A. Carretero

**Affiliations:** ^1^ Hypertension and Vascular Research DivisionDepartment of Internal MedicineHenry Ford HospitalDetroitMichigan; ^2^ Department of Public Health SciencesHenry Ford HospitalDetroitMichigan

**Keywords:** Ac‐SDKP, hypertension, lupus, NZBWF1

## Abstract

Systemic lupus erythematosus (SLE) is an autoimmune disease with a high prevalence of hypertension. NZBWF1 (SLE‐Hyp) mice develop hypertension that can be prevented by modulating T cells. The peptide *N*‐acetyl‐seryl‐aspartyl‐lysyl‐proline (Ac‐SDKP) decreases renal damage and improves renal function in a model of SLE without hypertension (MRL/*lpr*). However, it is not known whether Ac‐SDKP prevents hypertension in NZBWF1 mice. We hypothesized that in SLE‐Hyp, Ac‐SDKP prevents hypertension and renal damage by modulating T cells. Animals were divided into four groups: (1) control + vehicle, (2) control + Ac‐SDKP, (3) SLE + vehicle, and (4) SLE + Ac‐SDKP. Systolic blood pressure (SBP), albuminuria, renal fibrosis, and T‐cell phenotype were analyzed. SBP was higher in SLE compared to control mice and was not decreased by Ac‐SDKP treatment. Half of SLE mice developed an acute and severe form of hypertension accompanied by albuminuria followed by death. Ac‐SDKP delayed development of severe hypertension, albuminuria, and early mortality, but this delay did not reach statistical significance. Ac‐SDKP prevented glomerulosclerosis, but not interstitial fibrosis in SLE‐Hyp mice. SLE‐Hyp mice showed a decrease in helper and cytotoxic T cells as well as an increase in double negative lymphocytes and T helper 17 cells, but these cells were unaffected by Ac‐SDKP. In conclusion, Ac‐SDKP prevents kidney damage, without affecting blood pressure in an SLE animal model. However, during the acute relapse of SLE, Ac‐SDKP might also delay the manifestation of an acute and severe form of hypertension leading to early mortality. Ac‐SDKP is a potential tool to treat renal damage in SLE‐Hyp mice.

## Introduction

Systemic lupus erythematosus (SLE) is a severe autoimmune disease of unknown origin, the pathogenesis of which is incompletely understood. SLE has an unpredictable course and a wide spectrum of disease manifestations with remissions and relapses occurring over time. Hypertension is the major risk factor for the progression of renal, vascular, and heart disease. Individuals with SLE develop hypertension (Al‐Herz et al. 2003) and are at an alarmingly high risk for stroke, myocardial infarction, atherosclerosis, and renal disease (Manzi et al. 1997; Rahman et al. 2000; Bruce 2005; Ryan and McLemore 2007). Additionally, hematological abnormalities such as anemia, leucopenia, and lymphopenia are extremely common at the time of diagnosis and during the follow‐up course in SLE population (Aleem et al. 2014). Despite the severity of the disease, which often starts in the late childhood or adolescence and predominantly affects females in their reproductive years, treatment options are scarce and include immunosuppressive drugs, steroids, and monoclonal antibodies targeting B cells and B‐cell activating factor. A hallmark of the disease is the deposition of immune complexes in the tissues resulting in inflammation induction (Ohl and Tenbrock 2015). Effector/memory CD4^+^ helper T cells (Th) regulate the immune response by releasing of both proinflammatory and anti‐inflammatory cytokines. Based on effector function, Th cells are classified in two subsets: Th1 cells that release proinflammatory cytokines including interferon‐gamma (IFN‐*γ*), and Th2 cells that release interleukin (IL)‐4 that counteracts the IFN‐*γ* effects. Th17 cells release a unique IL‐17 cytokine involved in many autoimmune diseases, including SLE (Crispin and Tsokos 2009). By contrast, FoxP3^+^ regulatory T cells (T_regs_) are responsible for maintaining self‐tolerance by suppressing autoreactive lymphocytes. Alterations of T‐cell subtypes toward Th1 and Th17 phenotypes are thought to e important in autoimmune disease development. Female NZBWF1 (SLE‐Hyp) mice are the oldest classical model of SLE generated by the F1 hybrid between New Zealand Black (NZB) and New Zealand White (NZW) strains. Both NZB and NZW display limited autoimmunity, whereas NZBWF1 hybrids develop a severe phenotype comparable to that of lupus patients (Theofilopoulos and Dixon 1985). These lupus‐like phenotypes include lymphadenopathy, splenomegaly, elevated serum antinuclear autoantibodies, and immunocomplex‐mediated glomerulonephritis that becomes apparent at 20–24 weeks of age leading to kidney failure and death at 40–48 weeks of age (Theofilopoulos and Dixon 1985). Moreover, female SLE‐Hyp mice develop nonsalt‐sensitive hypertension (Mathis et al. 2011) and endothelial dysfunction at 34–36 weeks of age (Ryan and McLemore 2007). High blood pressure and albuminuria in NZBWF1 mice can be prevented by immunoregulatory drugs, such as etanercept (tumor necrosis factor‐alpha [TNF‐*α*] antagonist) (Venegas‐Pont et al. 2010), hydroxychloroquine (Gomez‐Guzman et al. 2014) or anti‐CD20 antibodies (Mathis et al. 2014), indicating that hypertension development in this particular model depends on an inflammatory immune mechanism.

*N*‐acetyl‐seryl‐aspartyl‐lysyl‐proline (Ac‐SDKP) is a naturally occurring tetrapeptide present in plasma, urine, and several tissues including the heart and kidney (Cavasin et al. 2007). Previously, we reported that Ac‐SDKP exerts anti‐inflammatory as well as immunoregulatory properties, and prevents hypertensive end‐organ damage in addition to autoimmune myocarditis and SLE (Peng et al. 2001; Rhaleb et al. 2001; Omata et al. 2006; Liao et al. 2010; Nakagawa et al. 2012; Gonzalez et al. 2014). Indeed, we and others have reported that in MRL/*lpr* mice, a model of SLE that differs from female NZBWF1, Ac‐SDKP treatment decreased lupus nephritis, renal inflammation, and fibrosis (Omata et al. 2006; Liao et al. 2015). However, in these previous studies, we could not study whether Ac‐SDKP prevents hypertension in SLE because the MRL/*lpr* model develops lupus nephritis without hypertension. Ac‐SDKP was initially described as a physiological inhibitor of hematopoietic stem cell proliferation (Lenfant et al. 1989). Ac‐SDKP is mainly hydrolyzed by angiotensin‐converting enzyme (ACE) (Azizi et al. 1997) and it has been suggested that chronic accumulation of Ac‐SDKP in plasma during ACE inhibition could participate in the alterations of hematopoiesis observed with ACE inhibitor treatment (Comte et al. 1997). Therefore, abnormalities in hematopoiesis such as anemia and leucopenia which are common in SLE, could be aggravated by Ac‐SDKP. Because hypertension development in SLE‐Hyp mice is caused particularly by autoimmune mechanisms, we hypothesized that Ac‐SDKP prevents development of autoimmune‐mediated kidney damage and hypertension without causing the adverse effects. Moreover, we hypothesized that the beneficial effects of Ac‐SDKP are mediated by the inhibition of T‐cell differentiation.

## Methods

### Experimental animals

Female NZBWF1 (SLE‐Hyp) and NZW (control) mice were obtained from Jackson Laboratories (Bar Harbor, ME). Animals were housed in an air‐conditioned room with a 1212‐h light‐dark cycle and given standard rodent chow (0.4% sodium) with tap water. Animals were allowed 7 days to adjust to their new environment. All protocols were approved by the Institutional Animal Care and Use Committee (IACUC) of Henry Ford Hospital. Before all surgical procedures, butorphanol (2 mg/kg sc) was used to induce analgesia and pentobarbital sodium (50 mg/kg ip) was used for anesthesia.

### Experimental protocols

#### Protocol 1 – study of the Ac‐SDKP effects on blood pressure, mortality, and renal damage in SLE

Female SLE‐Hyp mice and NZW (control) mice at 24–38 weeks of age were treated either with 0.9% sodium chloride containing 0.01 N acetic acid vehicle alone or with 800 *μ*g/kg per day Ac‐SDKP. The rats were divided into four groups: (1) control + vehicle (Ctl + Veh, *n* = 6), (2) control + Ac‐SDKP (Ctl + Ac‐SDKP, *n* = 6), (3) SLE‐Hyp + vehicle (SLE + Veh, *n* = 10), and (4) SLE‐Hyp + Ac‐SDKP (SLE + Ac‐SDKP, *n* = 10). Ac‐SDKP was infused subcutaneously at a dosage of 800 *μ*g/kg per day via an osmotic minipump (Alzet, Cupertino, CA) as described previously (Liao et al. 2015). Systolic blood pressure (SBP) was monitored by tail cuff measurements, whereas urinary albumin excretion was analyzed weekly by ELISA. At 38 weeks of age, animals were anesthetized with pentobarbital, killed, then plasma and organs were collected.

#### Protocol 2 – study of hematological parameters and T‐cell phenotypes

Female SLE‐Hyp and control mice were divided into four groups as described in protocol 1. Ac‐SDKP was infused subcutaneously at a higher dosage of 2400 *μ*g/kg per day via osmotic minipump from 20 to 35 weeks of age. We monitored body weight and performed standard hematologic studies at 32–33 weeks of age. Flow cytometry analysis was performed at 34–35 weeks of age to evaluate T‐cell phenotype.

### Systolic blood pressure

SBP was measured in conscious mice using a noninvasive computerized tail cuff system (MC‐4000, from Hatteras Instruments, Cary, NC) as described previously (Yang et al. 1999). Mice were trained for 3 days by measuring SBP daily, after which SBP was measured and recorded weekly. The results are expressed as the mean SBP ± standard error and also as % of incidence of severe hypertension (defined as SBP >140 mmHg).

### Urinary albumin excretion

The animals were placed in metabolic cages, and the urine was collected for 24 h. The urine samples were centrifuged twice at 13,400*g* at 4°C and the supernatant was filtered using a 0.22‐*μ*m low‐protein retention filters. Albumin concentration was measured by ELISA using a commercially available kit (GenWay Biotech., San Diego, CA). The 24‐h albumin excretion was calculated by multiplying the urine albumin concentration by the urine volume and expressed as *μ*g/24 h. The incidence of albuminuria (defined as a 24‐h albumin excretion rate >50 *μ*g) was monitored weekly.

### Glomerular matrix analysis

Paraffin‐embedded tissue sections (4 *μ*m) were stained with periodic acid Schiff (PAS). At least 35 glomeruli within randomly chosen fields of renal cortex were imaged at 400× magnification. A dark pink color was considered positive staining representing the extracellular matrix. Glomerular matrix was analyzed by computerized image analysis system (Microsuite Biological imaging software, Olympus America, Center Valley, PA) and positive staining was expressed as the percentage of glomerular area. All of the images shown in this study were captured and analyzed using the same imaging system. All measurements and analyses were performed in a blind fashion.

### Collagen deposition

Picrosirius red staining was used to quantify renal interstitial collagen deposition (Nakagawa et al. 2012; Liao et al. 2015). Randomly chosen fields within corticomedullary junctions were imaged at 200× magnification. The interstitial collagen fraction was calculated as the ratio of the collagen‐positive area to the imaging area.

### Flow cytometry analysis

Heparinized blood was collected from tail vein, and red blood cells (RBCs) were lysed using Ammonium‐Chloride‐Potassium (ACK) buffer. LIVE/DEAD Aqua dead cell staining kit (Life Technologies, Carlsbad, CA) was used to exclude dead cells. Cells were fixed and permeabilized using a commercially available buffer (eBioscience, San Diego, CA) and then Fc receptors blocked with an anti‐CD16/32 antibody. Cells were stained with fluorescein isothiocyanate (FITC) anti‐mouse CD4 (Biolegend, San Diego, CA, clone RM4‐4), alexa fluor 700 anti‐mouse CD8 (Biolegend, clone 53‐6.7), and phycoerythrin (PE) anti‐mouse/human FoxP3 (Biolegend, clone 150D) for 20 min. For intracellular cytokine staining, blood cells were stimulated with 50 ng/mL Phorbol 12‐Myristate 13 Acetate (Sigma Aldrich, St. Louis, MO), 1 *μ*g/mL ionomycin (Sigma Aldrich), and 5 *μ*g/mL brefeldin A (Sigma Aldrich) for 4 h at 37°C. Cells were fixed, permeabilized and stained with FITC anti‐mouse CD4, brilliant violet anti‐mouse IFN‐*γ* (Biolegend, clone XM 61.2), PE, anti‐mouse IL‐4 (Biolegend, clone 11B11), and alexa fluor 647, anti‐mouse IL‐17 (Biolegend, clone TC11‐18H10.1) antibodies to identify CD4^+^ IFN‐*γ* ^+^ Th1 cells, CD4^+^ IL‐4^+^ Th2 cells, and CD4^+^ IL‐17^+^ Th17 cells. At least 20,000 cells were analyzed using a BD Fortessa flow cytometer (BD Biosciences, San Jose, CA). Flow cytometry data were analyzed using FlowJo software (BD Biosciences).

### Hematological studies

Heparinized blood was collected from tail vein. The RBC and white blood cell (WBC) counts were determined using a hemocytometer with a Neubauer chamber. For the WBC count, blood samples were resuspended in 2% acetic acid and incubated for 1 min to lyse the RBC. For the differential WBC count, we prepared blood smears that were stained with Hema‐3 staining kit (Sigma Aldrich). Cells were quantified under a Nikkon Eclipse E600 microscope (Nikon, Tokyo, Japan). Neutrophil, lymphocyte, and monocyte cell lineages were identified by morphology and differential staining patterns. The absolute cell numbers were derived by multiplying the total WBC count by the percent of neutrophils, lymphocytes, or monocytes.

### Data analysis

All data are expressed as means ± SE. Statistical significance of the data was calculated by Kruskall–Wallis test followed by a Wilcoxon two‐sample analysis. A value of *P *<* *0.05 was considered significant.

## Results

### Effects of Ac‐SDKP on SLE disease severity

**Figure 1:**
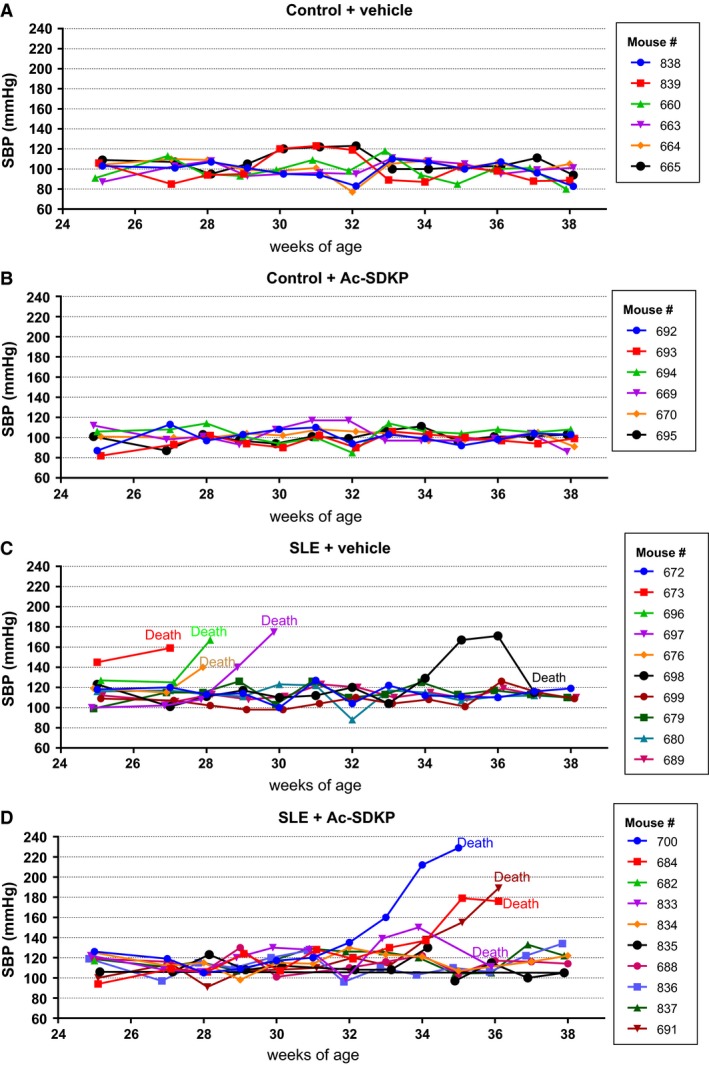
SBP measurements by tail cuff in (A) nonlupus mice treated with vehicle alone (control + vehicle, *n* = 6), (B) nonlupus mice treated with Ac‐SDKP (control + Ac‐SDKP,* n* = 6), (C) lupos mice treated with vehicle (SLE + vehicle, *n* = 10), and (D) lupus mice treated with Ac‐SDKP (SLE + Ac‐SDKP,* n* = 10). Blood pressure was monitored weekly from 25 to 38 weeks of age. Vehicle or Ac‐SDKP (800 *μ*g/kg per day) were infused via osmotic minipump from the age of 25 weeks. The recording of each individual mouse SBP is represented in different colors and the mouse ID number is shown at the figure inset. Some of the SLE mice developed a very aggressive and acute form of hypertension (SBP >140 mmHg) that was followed by death. The time of the manifestation of the acute hypertension manifestation in vehicle‐treated SLE‐Hyp mice occurred from 27 to 29 weeks of age, whereas in Ac‐SDKP‐treated animals, it occurred between 32 and 35 weeks of age. SBP, systolic blood pressure; Ac‐SDKP, *N*‐acetyl‐seryl‐aspartyl‐lysyl‐proline; SLE, systemic lupus erythematosus.

**Figure 2:**
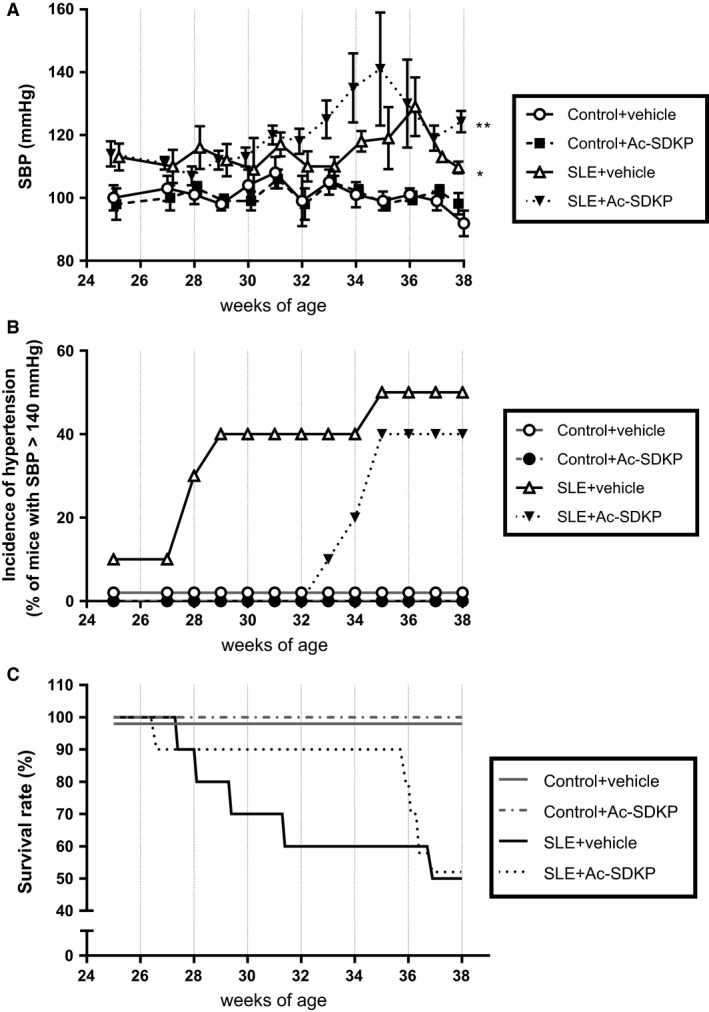
SBP was monitored by tail cuff method in control and SLE mice treated either with vehicle or Ac‐SDKP (800 *μ*g/kg per day) from 24 to 38 weeks of age. The results are expressed as (A) SBP mean ± standard error and (B) incidence of severe hypertension (defined as SBP >140 mmHg). (C) Effect of Ac‐SDKP and vehicle on survival rate in control and SLE mice from 25 to 38 weeks of age. The mortality and incidence of severe hypertension tended to be delayed in Ac‐SDKP‐treated SLE mice. *P <* *0.001 SLE + veh versus control + veh and **P <* *0.07 SLE + Ac‐SDKP versus SLE + veh. SBP, systolic blood pressure; SLE, systemic lupus erythematosus; Ac‐SDKP, *N*‐acetyl‐seryl‐aspartyl‐lysyl‐proline.

**Figure 3:**
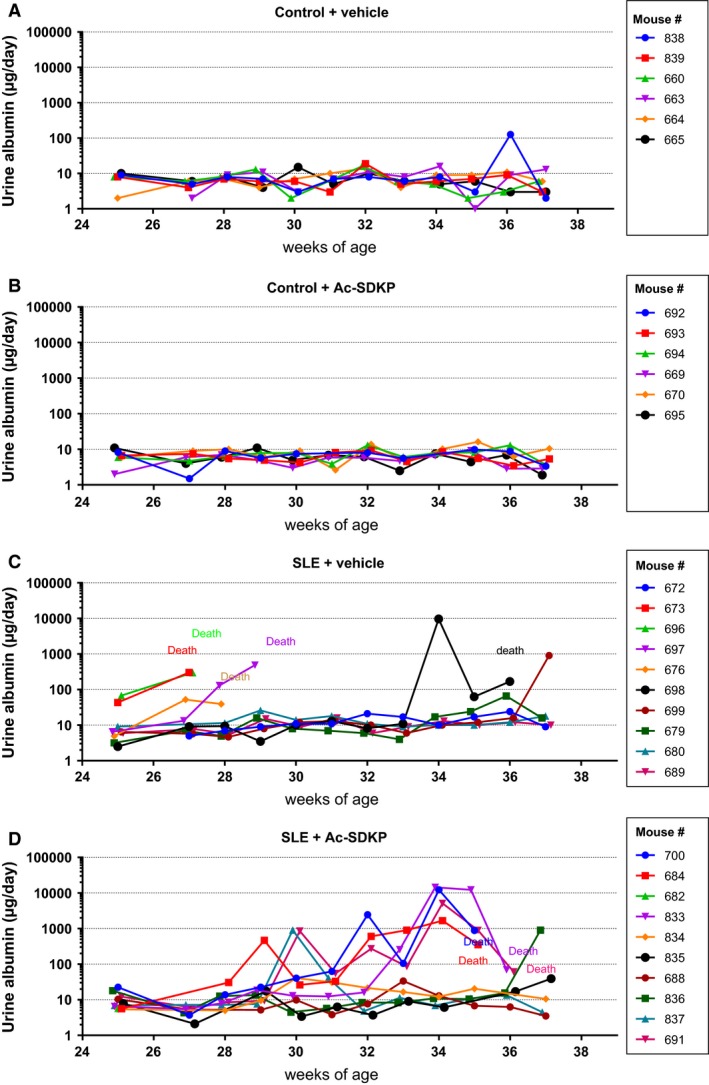
Albumin excretion rate measurements by ELISA in (A) control + vehicle (*n* = 6), (B) control + Ac‐SDKP (*n* = 6), (C) SLE + vehicle (*n* = 10), and (D) SLE + Ac‐SDKP (*n* = 10). The albumin excretion rate was monitored weekly from 25 to 38 weeks of age. Vehicle or Ac‐SDKP (800 *μ*g/kg per day) were infused via osmotic minipump from the age of 24 weeks. The recording of each individual mouse albumin excretion is represented in different colors, and the mouse ID number is shown in the figure inset. Some animals developed flares and remissions, whereas others did not show any indication of albuminuria. The time frame of albuminuria development in the SLE + veh group varied from 27 to 37 weeks of age. Ac‐SDKP tended to delay the development of massive albuminuria and death in SLE‐Hyp. Ac‐SDKP, *N*‐acetyl‐seryl‐aspartyl‐lysyl‐proline; SLE, systemic lupus erythematosus.

**Figure 4:**
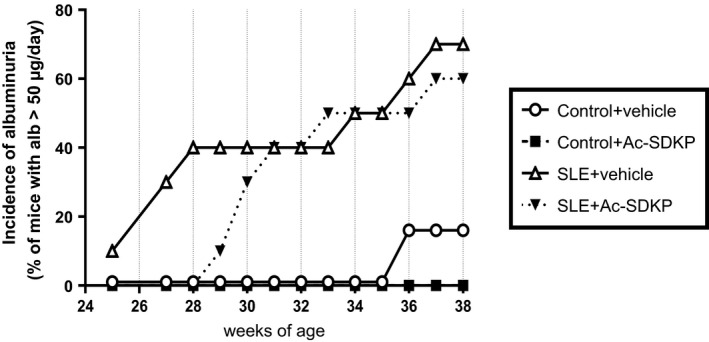
The albumin excretion rate was measured by ELISA and monitored in control and SLE with hypertension mice treated either with vehicle or Ac‐SDKP (800 *μ*g/kg per day) from 24 to 38 weeks of age. The results are expressed as incidence of albuminuria (defined as urinary albumin excretion >50 *μ*g/day). The incidence of albuminuria tended to be delayed in Ac‐SDKP‐treated SLE mice. SLE, systemic lupus erythematosus; Ac‐SDKP, *N*‐acetyl‐seryl‐aspartyl‐lysyl‐proline.

We observed a remarkable variability in both the manifestation time and the SP‐defined disease severity among the animals (Fig. 1). The SBP raw data values shown in Figure 1 were averaged and analyzed. When we compared the SBP averages among the groups, we observed a significantly higher SBP in SLE + veh mice compared to the nonlupus control group, and this increase was not prevented by Ac‐SDKP (Fig. 2A). In Figure 1C and B, we also observed that some SLE mice developed a severe and acute form of hypertension (SBP >140 mmHg) that was followed by death. Animals found dead contained an excessive amount of fluid in the abdominal cavity, whereas no bleeding in brain, chest, or abdominal cavity was found. Ac‐SDKP tended to delay the manifestation of severe hypertension in SLE‐Hyp by 6 weeks of age (Fig. 2B). However, these differences did not reach statistical significance due to the great variability observed in this model. The mortality rate in SLE‐Hyp mice was 50% at the end of the experimental protocol, whereas all of the animals in the control group survived. Although Ac‐SDKP treatment did not reduce the mortality rate, it tended to delay death in these animals (Fig. 2C). In control mice, Ac‐SDKP had no effect on the mortality rate. During the albuminuria follow‐up, some animals developed flares and remissions, whereas other animals showed no indication of SLE activity (Fig. 3). The time frame of albuminuria development in the SLE + veh group varied from 27 to 37 weeks of age. Seventy percent of SLE‐Hyp mice developed severe albuminuria (defined as a 24‐h albumin excretion rate >50 *μ*g), whereas no albuminuria was detected in control mice (Fig. 3). Ac‐SDKP tended to delay the development of severe albuminuria in SLE‐Hyp (Fig. 4).

**Figure 5:**
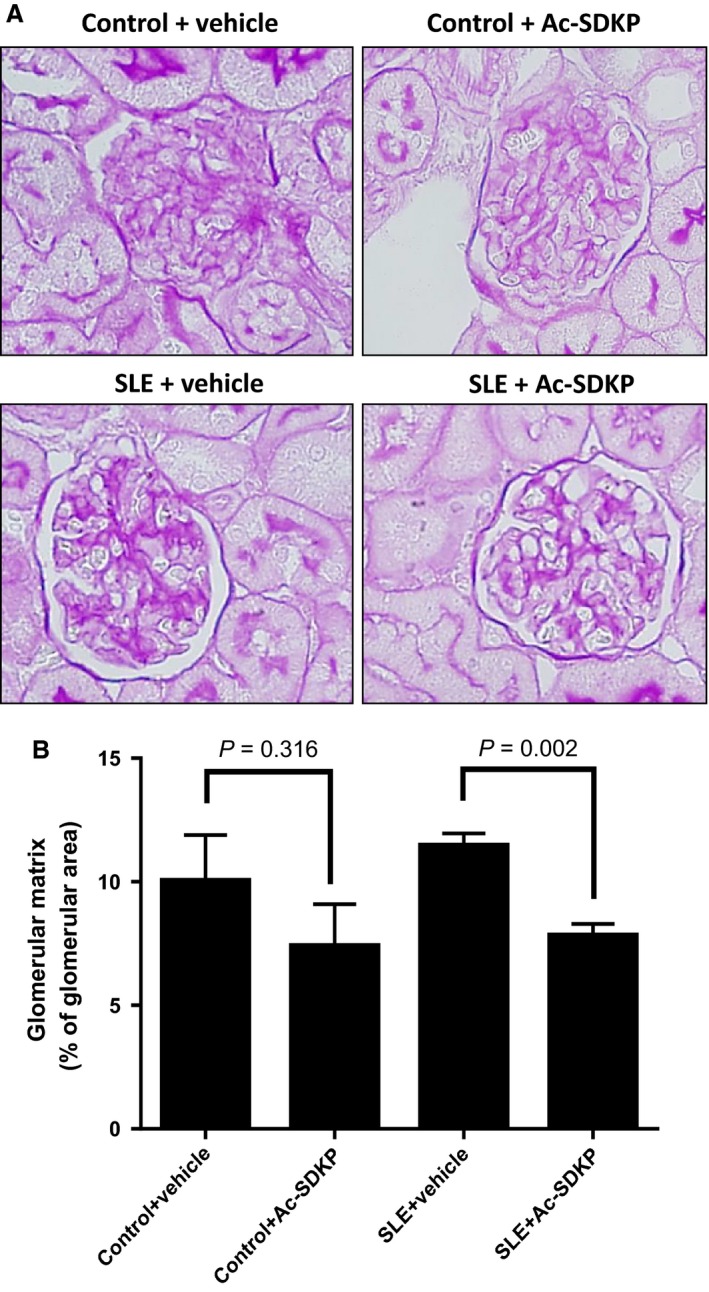
(A) Representative pictures of glomerular matrix deposition expressed as percentage of total glomerular area and measured by periodic acid Schiff staining in mice that survived at 38 weeks. (B) Quantitative data on glomerular matrix deposition. In SLE‐Hyp, Ac‐SDKP prevented glomerular matrix deposition indicating protection from renal damage. In control mice, Ac‐SDKP also tended to decrease glomerulosclerosis, but it did not reach statistical significance. SLE, systemic lupus erythematosus; Ac‐SDKP, *N*‐acetyl‐seryl‐aspartyl‐lysyl‐proline.

Histological analysis of the kidneys revealed that in SLE‐Hyp mice, Ac‐SDKP decreased glomerulosclerosis (SLE + veh: 11.5 ± 0.5 vs. SLE + Ac‐SDKP: 7.8 ± 0.5% of total glomerular area; Fig. 5) but not interstitial collagen deposition (SLE + veh: 4.6 ± 0.5 vs. SLE + Ac‐SDKP: 4.7 ± 0.5% of area; Table 1). No difference in kidney weight when corrected by body weight was observed between control and SLE‐Hyp mice (Ctl + veh: 5.8 ± 0.1 vs. SLE + veh: 5.6 ± 0.2; Table 1) and Ac‐SDKP had no effects on kidney weight. Plasma anti‐dsDNA IgG autoantibody titer, an index of disease activity, was measured at 38 weeks of age. We observed an important dispersion in anti‐dsDNA IgG antibody levels in control and SLE‐Hyp mice. SLE‐Hyp mice had higher anti‐dsDNA IgG levels compared to the control animals (SLE + veh: 137.5 ± 68.9 vs. Ctl + veh: 13.1 ± 2.8 kU/mL, *P* = 0.076), but this difference was not statistically significant due to the high standard deviation. In SLE‐Hyp mice, Ac‐SDKP treatment did not alter anti‐dsDNA antibody levels (SLE + veh: 137.5 ± 68.9 vs. SLE + Ac‐SDKP: 82.6 ± 25.3 kU/mL), nor body weight (SLE + veh: 41.3 ± 0.9 vs. SLE + Ac‐SDKP: 39.7 ± 1.5 g) (Table 1).

**Table 1:** Ac‐SDKP effects on body weight, anti‐dsDNA IgG, kidney weight, and interstitial collagen deposition in Ctl and SLE mice treated with veh or Ac‐SDKP (800 *μ*g/kg per day)

	Ctl + veh	Ctl + Ac‐SDKP	SLE + Veh	SLE + Ac‐SDKP
Body weight (g)	33.2 ± 1.0	32.5 ± 0.9	41.3 ± 0.9^a^	39.7 ± 1.5
Anti‐dsDNA IgG (kU/mL)	13.1 ± 2.8	93.4 ± 31.1	137.5 ± 68.9	82.6 ± 25.3
Kidney weight (mg)	194 ± 7	194 ± 9	228 ± 7	219 ± 10
Kidney weight corrected by body weight (mg/g)	5.8 ± 0.2	5.9 ± 0.2	5.6 ± 0.1	5.8 ± 0.3
Interstitial collagen deposition (% of area)	4.2 ± 0.5	4.0 ± 0.5	4.6 ± 0.4	4.7 ± 0.5

Values are means ± SE. Ac‐SDKP, *N*‐acetyl‐seryl‐aspartyl‐lysyl‐proline; Ctl, control; SLE, systemic lupus erythematosus; Veh, vehicle.

a *P < *0.01 Ctl + veh versus SLE + veh.

### Flow cytometry analysis of T cells

**Figure 6:**
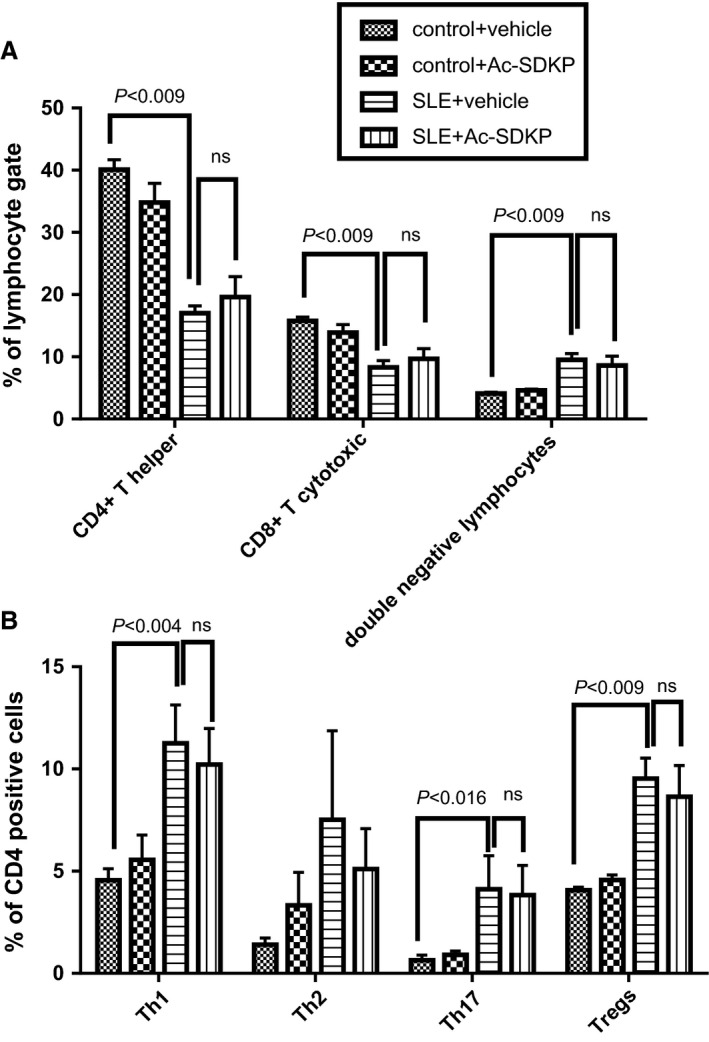
Flow cytometry analysis of peripheral blood cells sampled at 32–33 weeks of age in control and SLE with hypertension mice treated either with vehicle or Ac‐SDKP (2400 *μ*g/kg per day). (A) Percentage of CD4‐positive, CD8‐positive, and double negative lymphocytes. (B) Percentage of CD4‐positive Th1, Th2, T17 T cells, and T_regs_. SLE, systemic lupus erythematosus; Ac‐SDKP, *N*‐acetyl‐seryl‐aspartyl‐lysyl‐proline.

As shown in Figure 6A, the percentage of T helper (CD4^+^) and T cytotoxic (CD8^+^) lymphocytes was reduced in SLE‐Hyp mice in comparison to control mice. In contrast, the percentage of double negative CD4^−^ CD8^−^ lymphocytes (DN) were increased in SLE‐Hyp mice compared to the control animals. As shown in Figure 6B, intracellular cytokine analysis of peripheral blood T helper cells revealed an increase in the percentage of IFN‐*γ*‐positive Th1 cells in SLE‐Hyp mice compared to control mice. The percentages of both IL‐17‐positive Th17 cells and FoxP3‐positive Treg cells were also increased in SLE‐Hyp mice compared to control mice. Ac‐SDKP did not alter the percentage of peripheral blood T helper, cytotoxic T cells, or DN cells in control nor SLE‐Hyp (Fig. 6A). Intracellular flow cytometry analysis indicates that in SLE‐Hyp mice, Ac‐SDKP does not decrease the percentage of Th1 cells, Th17 cells, nor T_regs_ (Fig. 6B).

### Hematologic parameters

**Figure 7:**
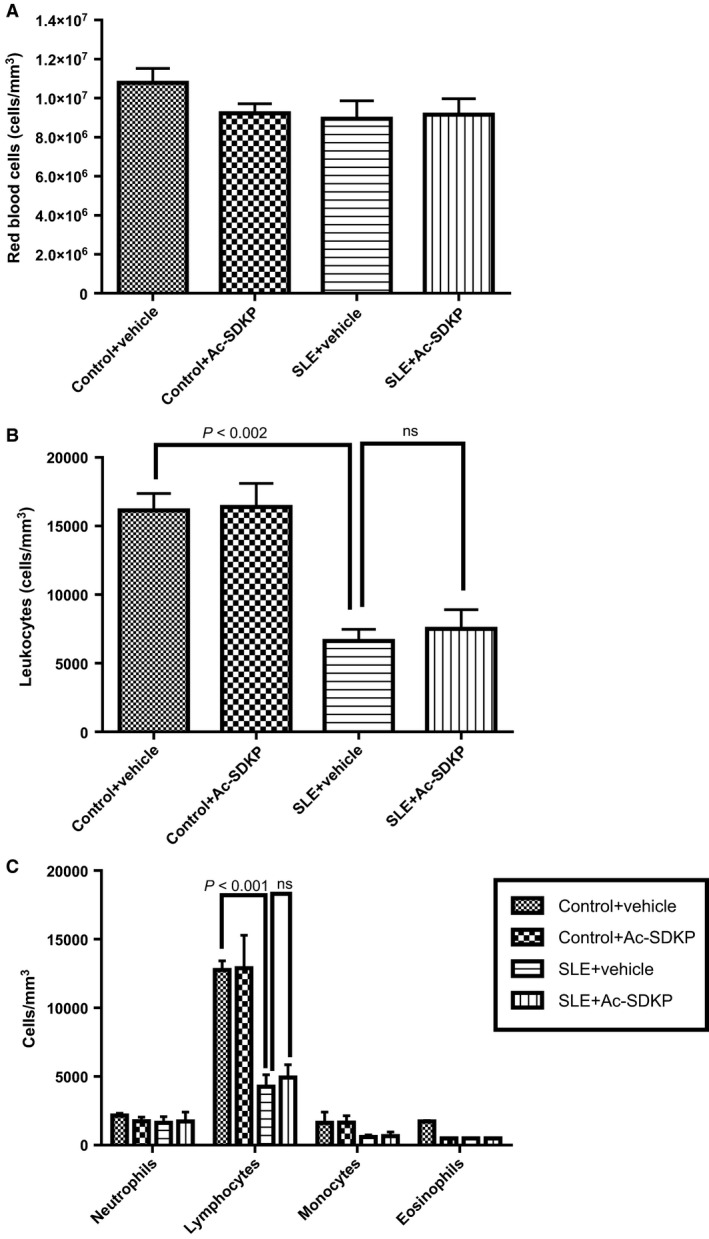
Hematologic studies performed at 32–33 weeks of age in control and SLE with hypertension mice treated either with vehicle or Ac‐SDKP (2400 *μ*g/kg per day). (A) Red blood cell total count expressed as number of cells per mm^3^, (B) white blood cell total count expressed as number of cells per mm^3^, and (C) white blood cell differential count expressed as number of cells per mm^3^. SLE, systemic lupus erythematosus; Ac‐SDKP, *N*‐acetyl‐seryl‐aspartyl‐lysyl‐proline.

As shown in Figure 7A, we did not observe differences in RBC count between control and SLE‐Hyp mice. However, the leukocyte count was significantly decreased in SLE‐Hyp mice compared to control mice (Fig. 7B). The differential leukocyte count in peripheral blood indicated that the leucopenia was caused by a severe decrease in lymphocytes, whereas differential counts of other WBCs such as neutrophils and monocytes, were not altered in SLE‐Hyp mice (Fig. 7C). Ac‐SDKP did not cause anemia in control mice or in SLE‐Hyp mice (Fig. 7A). Ac‐SDKP did not affect peripheral blood leukocyte counts in control mice or in SLE‐Hyp mice (Fig. 7B and C).

## Discussion

Previously, we and others reported that Ac‐SDKP prevents inflammation, fibrosis, lupus nephritis, and improves renal function in lupus‐prone MRL/*lpr* mice (Tan et al. 2012; Liao et al. 2015). MRL/*lpr* mice carry a genetic mutation in the lymphoproliferative (*lpr*) gene that encodes Fas ligand, and mice with inactive Fas ligand develop an aggressive form of lupus nephritis. However, MRL/*lpr* mice do not develop hypertension; thus, our previous study was limited on studying the effects of Ac‐SDKP on lupus nephritis, but not on the development of lupus‐associated hypertension development. Unlike MRL/*lpr* mice, female NZBWF1 (SLE‐Hyp) mice not only develop nephritis, but also hypertension. The etiology of the disease in SLE‐Hyp does not depend on a single genetic mutation. Here, we used the SLE‐Hyp as a model of hypertension related to SLE to test the effect of Ac‐SDKP on blood pressure and renal damage in SLE. From the beginning of the experimental protocol (25 weeks of age), SLE‐Hyp mice exhibited a significantly higher blood pressure, compared to control NZW mice. As shown in Figures 1, 3, we also observed that half of the animals in SLE‐Hyp mice developed acute flares with a fatal form of hypertension, massive albuminuria, and died immediately afterwards. The cause of death in these animals is likely renal and heart failure because the animals contained a large amount of fluids in the abdominal cavity, indicating ascites, whereas no hemorrhages or abnormalities were found in the chest, abdomen, or brain. Development of these fatal manifestations did not occur simultaneously in all animals, but on average, it occurred earlier than expected because the reported average lifespan for NZBWF1 is 35 weeks (245 days) (Dubois et al. 1966). We do not know the cause of these discrepancies, but we speculate that environmental factors such as diet, microbiome, environment temperature, and exposure to stressors could influence the lifespan of these animals. As shown in Figure 2B and C, the manifestation time of severe hypertension and mortality in vehicle‐treated SLE‐Hyp mice occurred from 27 to 29 weeks of age, whereas in Ac‐SDKP‐treated animals, it occurred between 32 and 35 weeks of age. Animals that survived were killed at 38 weeks and glomerular matrix deposition was measured (Fig. 5). There was a decrease in glomerular matrix deposition in Ac‐SDKP‐treated group indicating that Ac‐SDKP could be delaying the progression of kidney injury. Interestingly, SLE‐Hyp mice had a similar degree of glomerulosclerosis compared to control animals. We attribute these unexpected results to two factors. First, the animals with the highest degree of renal damage developed fatal renal disease and died before the end of the protocol. Thus, we only measured glomerular matrix deposition in the survivors that exhibited mild disease. Second, the control NZW mice do also develop a mild and limited SLE‐like disease during more advanced age. Thus, we expected some degree of glomerulosclerosis in the control group. Nevertheless, we observed a decrease in the degree of glomerular damage in Ac‐SDKP‐treated animals, and this is consistent with many previous studies (Peng et al. 2001; Rhaleb et al. 2011; Liao et al. 2015; Worou et al. 2015). Although Ac‐SDKP tended to delay the incidence of these fatal manifestations. The mean SBP value between the vehicle‐treated SLE‐Hyp mice and Ac‐SDKP‐treated mice was similar (Fig. 2A). One of the limitations of this study is the lack of renal functional analysis. In the nonhypertensive mice, Ac‐SDKP prevented the decline of renal function. Thus, future studies will needed to determine whether Ac‐SDKP improves renal function in SLE‐Hyp. It is not surprising that Ac‐SDKP does not decrease blood pressure in SLE because we previously showed that in many models of hypertension, Ac‐SDKP decreased renal inflammation and fibrosis without decreasing the blood pressure (Rhaleb et al. 2001; Gonzalez et al. 2014). Ryan et al. showed that the inflammatory response is involved in SLE‐associated hypertension development (Mathis et al. 2014). Because Ac‐SDKP is a potent anti‐inflammatory peptide, one might expect that Ac‐SDKP could prevent hypertension development in SLE or other immune‐mediated diseases. In this study, Ac‐SDKP decreased glomerular damage but without decreasing blood pressure, indicating that hypertension in SLE is does not result from glomerosclerosis. Petrin et al. (1993) reported a dissociation between arterial blood pressure and glomerulonephritis in SLE. Additionally, a lack of association between arterial blood pressure and nephritis in MRL/*lpr* mice [ref] supports the hypothesis that hypertension occurs independently of nephritis in SLE. In SLE‐Hyp, we observed many alterations in the proportion of T‐cell subsets. As shown in Figure 6A, we observed that in SLE‐Hyp mice had lower proportions of CD4^+^ T helper lymphocytes and CD8^+^ cytotoxic T lymphocytes, whereas CD4^−^ CD8^‐^ DN cells were augmented. Previous reports showed an expansion of DN T cells in lupus patients. DN T cells release IL‐1*β*, IL‐17, and IFN‐*γ* (Crispin and Tsokos 2009), provide help to B cells, and contribute to the abnormal autoantibody profile in lupus patients (Shivakumar et al. 1989). Additionally, SLE‐Hyp mice showed an increased proinflammatory Th1 and Th17 profile. Schmidt et al. (2015) and other investigators demonstrated that neutralizing IFN‐*γ* attenuates disease severity in SLE‐Hyp and MRL*/lpr* mice, whereas the Th17/IL‐17 immune response is not involved in the immunopathogenesis of lupus nephritis in MRL/*lpr* or SLE‐Hyp mice. The effects of IFN‐*γ* and IL‐17 on hypertension development in SLE‐Hyp mice remain unknown. Defects in or lack of T_regs_ are thought to contribute to SLE pathogenesis (Scheinecker et al. 2010). Paradoxically, we observed that the percentage of peripheral blood FoxP3^+^ T_regs_ were increased in SLE‐Hyp compared to controls. It has been reported previously that T_regs_ are suppressed in the spleen of SLE‐Hyp mice, but augmented in the lymph nodes and kidney of the same animals (Ohl and Tenbrock 2015). Our study is the first reporting T_reg_ levels in the peripheral blood of SLE‐Hyp mice. We speculate that the abundant T_regs_ observed in SLE‐Hyp might display low or absent suppressive effects as suggested by Ohl and Tenbrock (2015). Indeed, Zhou et al. (2009) suggested that FoxP3 instability in T_regs_ leads to pathogenic cells that release inflammatory cytokines. In MRL/*lpr* mice, Ac‐SDKP has been shown to decrease many proinflammatory factors such as the complement system activation (C5a), TNF‐*α*, RANTES, monocyte chemoattractant protein‐5 (MCP‐5), intercellular adhesion molecule‐1 (ICAM‐1), and the proinflammatory transcription factor NF‐kB. Most of the anti‐inflammatory effects of Ac‐SDKP are mainly mediated by its effects on the innate immunity (macrophages) (Sharma et al. 2008). However, whether Ac‐SDKP modulates T‐cell phenotype in SLE‐Hyp was still unknown. In this study we hypothesized that by shifting the balance of T cells toward an anti‐inflammatory phenotype Ac‐SDKP ameliorates the severity of the disease and decreases blood pressure in SLE. Contrary to our hypothesis, Ac‐SDKP did not balance T cells toward an anti‐inflammatory phenotype. We speculate that drugs targeting the T‐cell response and IL‐17 signaling could be beneficial not only to treat SLE, but also to treat SLE hypertension. Ac‐SDKP is suspected to be responsible for the anemia caused by ACE inhibitors (Abu‐Alfa and Perazella 2002), and SLE is a disease associated with hematological alterations such as anemia, lymphopenia, and thrombocytopenia (Aleem et al. 2014). Thus, a possible side effect of Ac‐SDKP on hematopoietic system could be expected in SLE. In SLE‐Hyp mice, the WBCs were dramatically decreased. Differential analysis revealed a severe lymphopenia, whereas no alterations were found in the numbers of neutrophils or monocytes. Ac‐SDKP did not correct these abnormalities, but did not further decrease the lymphocyte counts nor cause anemia in these animals, indicating Ac‐SDKP could be used to treat SLE without adverse effects.

We conclude that Ac‐SDKP prevents renal damage in SLE‐Hyp mice, ameliorating fatal renal disease manifestations and delaying mortality, without causing or aggravating anemia and lymphopenia. However, Ac‐SDKP fails to reduce blood pressure and systemic T‐cell phenotype. This study supports our previous findings indicating that Ac‐SDKP could be a potential therapeutic tool for preventing or treating renal damage in hypertension or other autoimmune diseases without affecting blood pressure.

## Conflict of Interest

None declared.
